# HbA_1c_ and Risks of All-Cause and Cause-Specific Death in Subjects without Known Diabetes: A Dose-Response Meta-Analysis of Prospective Cohort Studies

**DOI:** 10.1038/srep24071

**Published:** 2016-04-05

**Authors:** Guo-Chao Zhong, Ming-Xin Ye, Jia-Hao Cheng, Yong Zhao, Jian-Ping Gong

**Affiliations:** 1 Department of Hepatobiliary Surgery, The Second Affiliated Hospital of Chongqing Medical University, Chongqing 400010, China; 2 Department of Urinary Surgery, The Second Affiliated Hospital of Chongqing Medical University, Chongqing 400010, China; 3 School of Public Health and Management, Chongqing Medical University, Chongqing 400016, China

## Abstract

Whether HbA_1c_ levels are associated with mortality in subjects without known diabetes remains controversial. Moreover, the shape of the dose–response relationship on this topic is unclear. Therefore, a dose–response meta-analysis was conducted. PubMed and EMBASE were searched. Summary hazard ratios (HRs) were calculated using a random-effects model. Twelve studies were included. The summary HR per 1% increase in HbA_1c_ level was 1.03 [95% confidence interval (CI) = 1.01–1.04] for all-cause mortality, 1.05 [95% CI = 1.02–1.07) for cardiovascular disease (CVD) mortality, and 1.02 (95% CI = 0.99–1.07) for cancer mortality. After excluding subjects with undiagnosed diabetes, the aforementioned associations remained significant for CVD mortality only. After further excluding subjects with prediabetes, all aforementioned associations presented non-significance. Evidence of a non-linear association between HbA_1c_ and mortality from all causes, CVD and cancer was found (all *P*_non-linearity_ < 0.05). The dose–response curves were relatively flat for HbA_1c_ less than around 5.7%, and rose steeply thereafter. In conclusion, higher HbA_1c_ level is associated with increased mortality from all causes and CVD among subjects without known diabetes. However, this association is driven by those with undiagnosed diabetes or prediabetes. The results regarding cancer mortality should be treated with caution due to limited studies.

HbA_1c_, a major component of hemoglobin-glucose adducts^1^, reflects the average blood glucose level within the prior 2 to 3 months^2^, and is a well accepted biomarker for glycemic management in diabetic patients over the past several decades. HbA_1c_ has been consistently recommended as a diagnostic biomarker for diabetes by several societies^3^,^4^,^5^. At present, in clinical practice, a variety of diagnostic assays, including ion-exchange chromatography, electrophoresis, immunoassay, are available to measure HbA_1c_ levels^6^,^7^. Nonetheless, mounting evidence shows that electrochemical HbA_1c_ sensors (e.g., amperometric nonenzymatic sensors)^8^ and optical HbA_1c_ sensors (e.g., vibrational spectroscopy)^6^,^9^ possibly provide more convenient and cheaper assays for determining HbA_1c_ levels.

Previous studies in diabetic patients demonstrated a significant association between HbA_1c_ levels and all-cause mortality^10^, which presented a J-shaped pattern^11^. However, whether these findings can extend into subjects without known diabetes is largely unknown. To fill this gap, some epidemiological studies were conducted but presented inconsistent results. Several studies observed that high HbA_1c_ levels (≥6.5%, 48 mmol/mol) were significantly associated with increased all-cause mortality^2^,^12^,^13^, whereas others failed to find any significant associations with all-cause mortality across the whole HbA_1c_ range^14^,^15^. In addition, for the shape of the dose–response relationship between HbA_1c_ levels and mortality, previous studies have suggested a J-shaped pattern^12^,^15^, a U-shaped pattern^2^, and a linear pattern^13^. Although a 2011 meta-analysis^16^, including 5 studies, investigated the association of HbA_1c_ levels with mortality from cardiovascular disease (CVD) in non-diabetic population, substantial heterogeneity (*I*^*2*^ = 98.5%) and combination data from cross-sectional study^17^ with those from prospective studies^18^,^19^,^20^,^21^ raised concerns for the reliability of its findings.

With more attention to HbA_1c_ currently, investigation of the dose–response relationship between HbA_1c_ and mortality is critical for a better understanding of this biomarker, and can extend its’ application into a broader field. However, to the best of our knowledge, a comprehensive dose–response meta-analysis on the HbA_1c_-mortality association is not available to date. Therefore, the objectives of our study were to clarify the associations between HbA_1c_ levels and risks of death from all causes, CVD, or cancer in subjects without known diabetes, and to further investigate the exact shape of these associations.

## Methods

### Search strategy

We conducted this study and reported corresponding results in adherence to the PRISMA statement^22^. A comprehensive electronic search of PubMed and EMBASE was conducted up to January 2015. Detailed search strategy is presented in the Supplementary List S1. For including additional citations, we performed a manual search of the reference lists of included articles and pertinent reviews. We contacted the original authors to obtain extra information if necessary.

### Study selection

Studies were eligible for inclusion if they met the following criteria: (1) participants: subjects without known diabetes; (2) exposure: HbA_1c_ levels measured by methods standardized by the National Glycohemoglobin Standardization Program or International Federation of Clinical Chemistry^23^; (3) outcome: adjusted risk estimates for at least three quantitative HbA_1c_ categories on the associations of HbA_1c_ levels with risks of death from all causes, CVD, or cancer; (4) study design: prospective cohort study. We excluded studies in which subjects were free of diabetes but suffered from other conditions (e.g., acute coronary syndrome).

### Data extraction

Two investigators (G.C.Z. and J.H.C.) independently performed study screening through a two-stage method. At the first stage, we scrutinized titles and abstracts to exclude apparently irrelevant studies. At the second stage, we read carefully the full text to further exclude unrelated studies. Two investigators discussed together until consensus was reached when any discrepancy regarding the eligibility of studies occurred.

One investigator (G.C.Z.) performed data extraction, and then another investigator (J.H.C.) checked the results for accuracy. We re-checked data and discussed together to deal with any inconsistent results. Following information was extracted: first author, publication year, study location, mean age at baseline, sample size, mean follow-up duration, assessments of non-diabetic statuses and outcomes, methods of measuring HbA_1c_, deaths and person-years for each HbA_1c_ level, categories of HbA_1c_, maximally adjusted risk estimates and corresponding 95% confidence intervals (CIs), and adjustment factors. We extracted information from the publication with the longest follow-up duration when multiple publications derived from the same cohort.

### Data synthesis and analysis

In this meta-analysis, hazard ratio (HR) for every 1% increase in HbA_1c_ level was used to assess the HbA_1c_-mortality association. To support the International Federation of Clinical Chemistry HbA_1c_ system^23^, we also calculated HR for a 10 mmol/mol increase in HbA_1c_ level. For one study^24^, relative risk was treated as equivalent to HR. For another study^25^ whose authors reported risk estimates for men and women separately, we pooled these data to yield an overall estimate using a random-effects model. The Hedges Q statistic was employed to qualitatively assess heterogeneity across studies, with *P* < 0.10 indicating statistically significant heterogeneity. The *I*^*2*^ statistic was employed to quantify heterogeneity^26^, with *I*^*2*^ > 50% representing substantial heterogeneity, 30% ≤ *I*^*2*^ ≤ 50% representing moderate heterogeneity, and *I*^*2*^ < 30% representing low heterogeneity^27^.

We performed a two-stage dose–response meta-analysis to identify whether higher HbA_1c_ level was significantly associated with increased mortality. First, for the purpose of pooling risk estimates from included studies using different HbA_1c_ categorization, we employed the generalized least square regression described by Orsini and colleagues^28^ to calculate study-specific slopes (linear trends) and 95% CIs for every 1% increase in HbA_1c_ level within each study from the natural logs of maximally adjusted HRs and CIs across categories of HbA_1c_. Then, we pooled them using a random-effects model to obtain the summary risk estimates. This method is based on specific HbA_1c_ level, distribution of deaths and person-years, and adjusted risk estimates and 95% CIs. Since all included studies reported HbA_1c_ level as range, we designated the midpoint of lower and upper limits as the assigned level. When the highest range was open-ended, we calculated the assigned level by adding the width of the adjacent range to the highest value specified. When the lowest range was open-ended, we calculated the assigned level by subtracting half of the width of the adjacent range from the lowest value specified^29^. The method described by Hamling and colleagues^30^ was employed to convert risk estimates if the reference group reported in the original study was not the lowest group. For 4 studies^15^,^19^,^31^,^32^ whose authors did not report person-years for each HbA_1c_ category, we approximately estimated these data from mean follow-up duration and number of subjects. For studies that provided risk estimates with more than one level of adjustment, we calculated study-specific slopes and 95% CIs with minimally adjusted data to examine whether the observed associations were largely explained by confounding. To determine whether increased mortality was due to inclusion of subjects with undiagnosed diabetes (HbA_1c_ ≥ 6.5%, 48 mmol/mol), we repeated our meta-analyses by excluding these subjects. To investigate whether HbA_1c_ was still associated with mortality in subjects with normal HbA_1c_ level (HbA_1c_ < 5.7%, 39 mmol/mol), we repeated our meta-analyses by further excluding subjects with prediabetes (5.7% ≤ HbA_1c_ ≤ 6.4%, 39 mmol/mol ≤ HbA_1c_ ≤ 46 mmol/mol).

We explored a potential non-linear dose–response relationship between HbA_1c_ levels and mortality using restricted cubic spline function with 3 knots at the 10th, 50th, and 90th percentiles^33^,^34^. A *P*_non-linearity_ was obtained by testing the null hypothesis that the estimated value of the second spline equals zero^34^.

To determine the stability of summary results, we performed sensitivity analyses for all-cause and CVD mortality through three techniques, namely ignoring a single study in turn, repeating meta-analyses by a fixed-effects model, and applying various eligibility criteria. To identify whether the association of HbA_1c_ levels with mortality was modified by age, follow-up duration, sample size, and study location, we conducted subgroup analyses stratified by these study-level characteristics. A *P*_interaction_ between subgroups was calculated by meta-regression. Considering limited studies for CVD and cancer mortality, subgroup analyses were performed only for all-cause mortality.

Begg rank correlation test^35^ and Egger linear regression test^36^ were employed to test publication bias, with *P* < 0.1 indicating publication bias. We conducted all data analyses through STATA software (version12.0, StataCorp, College Station, TX). Statistical significance level was set at *P* < 0.05 under two-sided test unless otherwise specified.

## Results

### Study identification and selection

**Figure 1:**
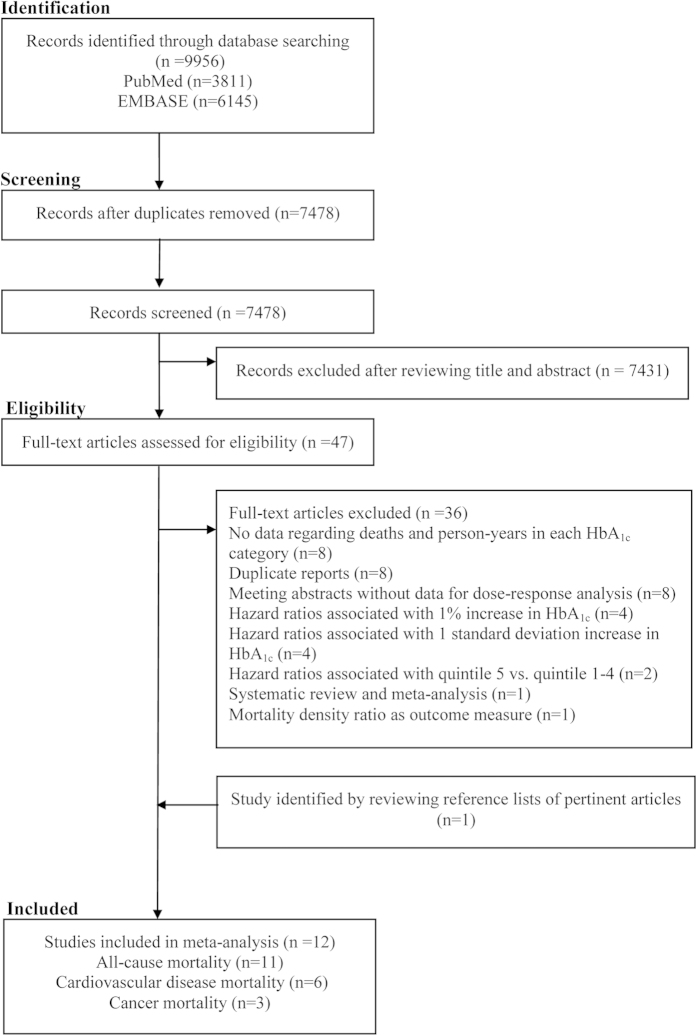
The flowchart of identifying relevant studies.

Our comprehensive retrieval identified 3,811 and 6,145 records from PubMed and EMBASE, respectively. After removing duplicates and excluding obviously unrelated records, there were remaining 47 records that were potentially relevant. After checking the full text, 36 were further excluded (detailed reasons for exclusion are shown in Fig. 1). We included two studies^25^,^37^ derived from the same cohort, because they reported different outcomes (one reported all-cause mortality^37^, another reported cancer mortality)^25^. In addition, we added one study^15^ through the handsearch. Thus, 12 studies (11 cohorts) were eligible for inclusion (Fig. 1).

### Study characteristics

The characteristics of included studies are summarized in Supplementary Table S1. The HbA_1c_ level ranged from 3.5% (15 mmol/mol)^24^ to 10.4% (90 mmol/mol)^24^ across studies. Of included studies, ten provided data necessary to calculate proportions of subjects in three clinical HbA_1c_ ranges (i.e., normal, prediabetes, and undiagnosed). Specifically, proportions of subjects with normal HbA_1c_ level, prediabetes, and undiagnosed diabetes were 85.90%, 11.33%, and 2.77%, respectively. The included studies were published between 2005^32^ and 2015^2^. Four studies were conducted in Europe^2^,^19^,^31^,^38^, 5 in the USA^14^,^15^,^24^,^25^,^37^, and remaining 3 in Asia^12^,^13^,^32^. The mean age of participants at baseline varied from 44.7 years^2^ to 78.7 years^19^ across studies. The sample size of included study ranged from 810^14^ to 26,549^24^. Our study included a total of 114,102 subjects, consisting of 41,616 men (36.5%) and 72,486 women (63.5%). The mean follow-up duration changed from 5.0 years^19^ to 14.2 years^14^, and during 1,161,714 person-years of follow-up, there were a total of 11,301 deaths. Most studies relied on self-reports of participants for the ascertainment of non-diabetic status, and only 2 studies^14^,^38^ confirmed it through diagnostic test or use of anti-diabetic medications. Of included studies, the information regarding vital status and causes of death was obtained from diverse sources, including death certificate^19^,^25^,^31^,^32^,^37^ and death registry^2^,^12^,^38^. Ten studies used high performance liquid chromatography method to measure HbA_1c_ level, and remaining 2 studies adopted affinity column method^14^ or turbidimetric immunoinhibition assay method^24^.

### Association of HbA_1c_ levels with all-cause mortality

**Figure 2:**
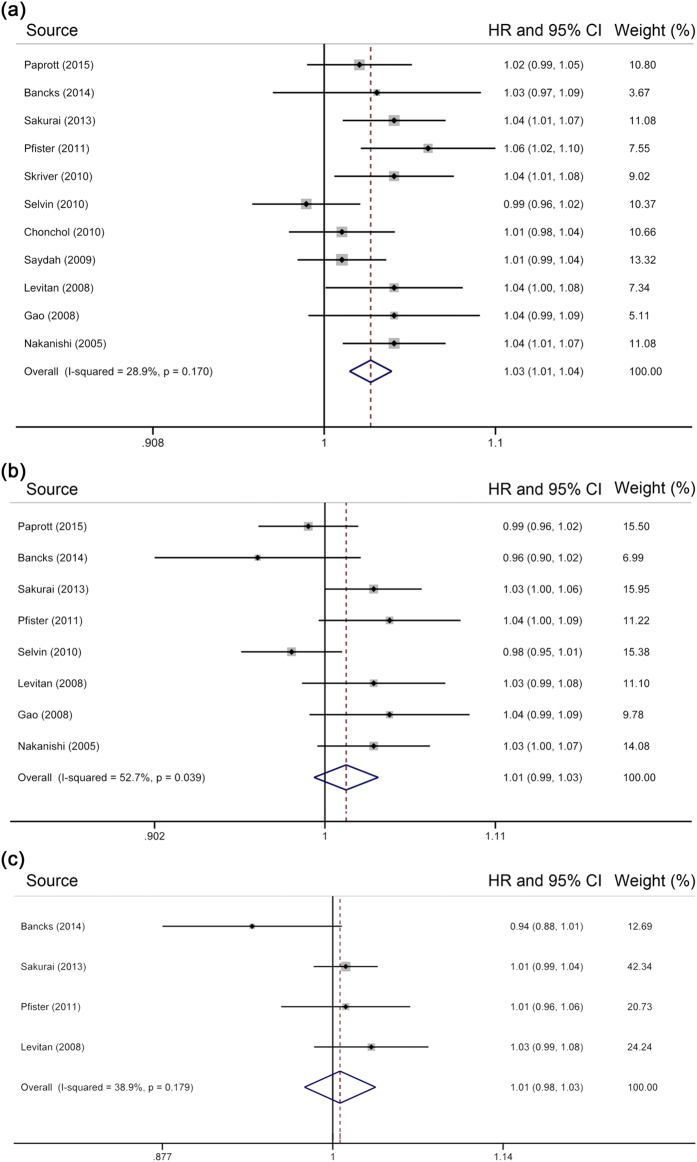
Meta-analysis on HbA_1c_ and all-cause mortality in (**a**) subjects without known diabetes, (**b**) those without diabetes, and (**c**) those with normal HbA_1c_ range. The squares represent the hazard ratio per 1% increase in HbA_1c_ level for each individual study, with the area reflecting the weight assigned to the study. The horizontal line across each square represents the 95% confidence interval. The diamond represents the summary hazard ratio per 1% increase in HbA_1c_ level, with width representing 95% confidence interval.

Eleven individual studies were eligible for the two-stage dose–response analysis of HbA_1c_ levels and all-cause mortality, involving a sum of 113,526 subjects and 11,301 deaths. The summary HR per 1% increase in HbA_1c_ level was 1.03 [95% CI = 1.01–1.04] (for every 10 mmol/mol increase, 1.03 [95% CI = 1.01–1.04]), with evidence of low heterogeneity (*I*^*2*^ = 28.9%, *P* = 0.17) (Fig. 2a). Parallel analysis with minimally adjusted data from 8 individual studies^2^,^13^,^14^,^15^,^24^,^31^,^32^,^37^ produced an HR of 1.03 [95% CI = 1.02 –1.05]. However, the initial pooled result presented non-significance after excluding participants with undiagnosed diabetes [HR = 1.01, 95% CI = 0.99–1.03] (Fig. 2b)^2^,^12^,^13^,^19^,^24^,^31^,^32^,^37^. After further excluding subjects with prediabetes, a non-significant risk estimate was observed [HR = 1.01, 95% CI = 0.98–1.03] (Fig. 2c)^12^,^13^,^24^,^25^.

### Association of HbA_1c_ levels with CVD mortality

**Figure 3:**
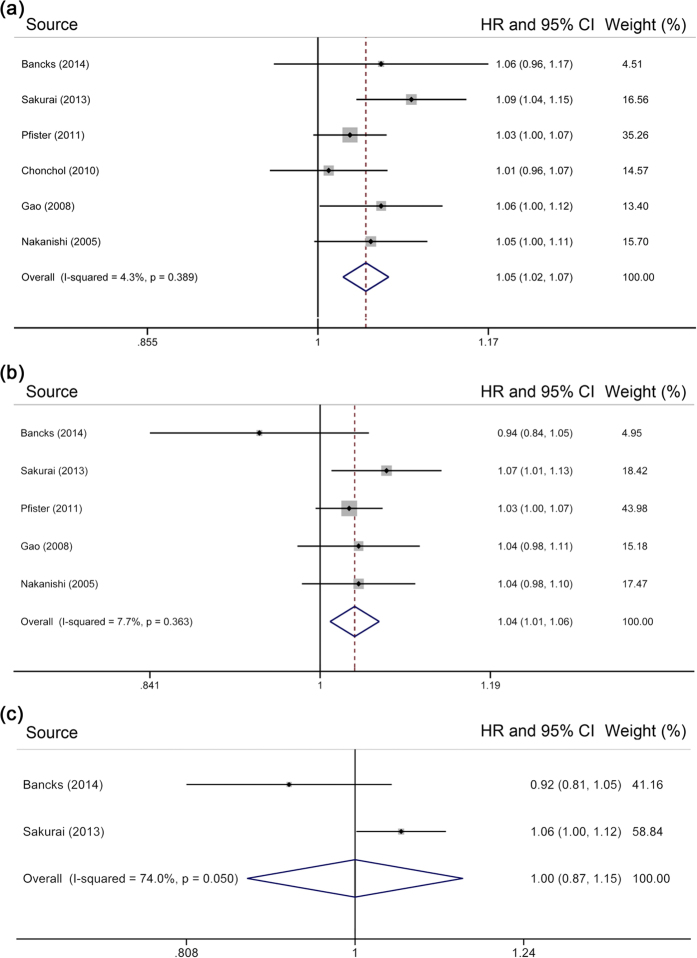
Meta-analysis on HbA_1c_ and cardiovascular disease mortality in (**a**) subjects without known diabetes, (**b**) those without diabetes, and (**c**) those with normal HbA_1c_ range. The squares represent the hazard ratio per 1% increase in HbA_1c_ level for each individual study, with the area reflecting the weight assigned to the study. The horizontal line across each square represents the 95% confidence interval. The diamond represents the summary hazard ratio per 1% increase in HbA_1c_ level, with width representing 95% confidence interval.

Six individual studies were eligible for the association between HbA_1c_ levels and CVD mortality, with a total of 36,695 participants and 1,713 deaths. The pooled HR per 1% increase in HbA_1c_ level was 1.05 [95% CI = 1.02–1.07] (for every 10 mmol/mol increase, 1.04 [95% CI = 1.02– 1.06]) (Fig. 3a), with evidence of low heterogeneity (*I*^*2*^ = 4.3%, *P* = 0.39). Meta-analysis of HRs with the least degree of adjustment^13^,^14^,^31^,^32^ yielded an HR of 1.04 [95% CI = 1.01–1.07]. After excluding participants with undiagnosed diabetes^12^,^13^,^19^,^31^,^32^, the result was attenuated slightly, but remained significant [HR = 1.04, 95% CI = 1.01–1.06] (Fig. 3b). However, after further excluding participants with prediabetes^12^,^13^, higher HbA_1c_ level was not significantly associated with increased mortality from CVD [HR = 1.00, 95% CI = 0.87–1.15] (Fig. 3c).

### Association of HbA_1c_ levels with cancer mortality

**Figure 4:**
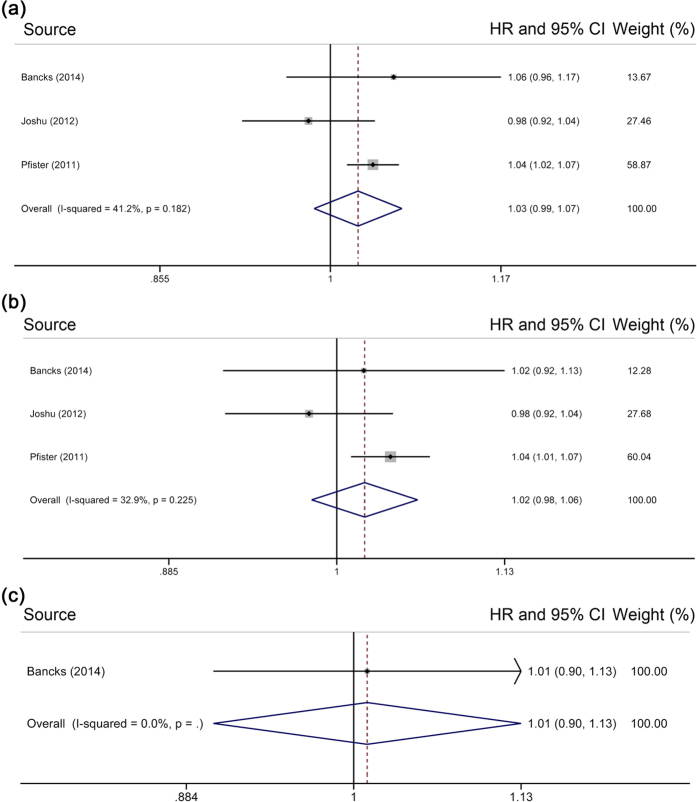
Meta-analysis on HbA_1c_ and cancer mortality in (**a**) subjects without known diabetes, (**b**) those without diabetes, and (**c**) those with normal HbA_1c_ range. The squares represent the hazard ratio per 1% increase in HbA_1c_ level for each individual study, with the area reflecting the weight assigned to the study. The horizontal line across each square represents the 95% confidence interval. The diamond represents the summary hazard ratio per 1% increase in HbA_1c_ level, with width representing 95% confidence interval.

Only 3 studies were included in the two-stage dose–response analysis of HbA_1c_ levels and cancer mortality, involving a total of 36,252 individuals and 2,115 deaths. Random-effects meta-analysis revealed an HR of 1.03 [95% CI = 0.99–1.07] per 1% increase in HbA_1c_ level (for every 10 mmol/mol increase, 1.02 [95% CI = 0.98–1.06]) (Fig. 4a), with evidence of moderate heterogeneity (*I*^*2*^ = 41.2%, *P* = 0.18). With 2 studies^25^,^31^ reporting minimally adjusted HRs, a pooled HR of 1.04 [95% CI = 1.01–1.06] was obtained. Our analysis produced a non-significant pooled risk estimate [HR = 1.02, 95% CI = 0.98–1.06] (Fig. 4b) after excluding participants with undiagnosed diabetes^12^,^25^,^31^. Further excluding participants with prediabetes^12^ resulted in an HR of 1.01 [95% CI = 0.90–1.13] (Fig. 4c).

### Non-linear dose–response analyses

**Figure 5:**
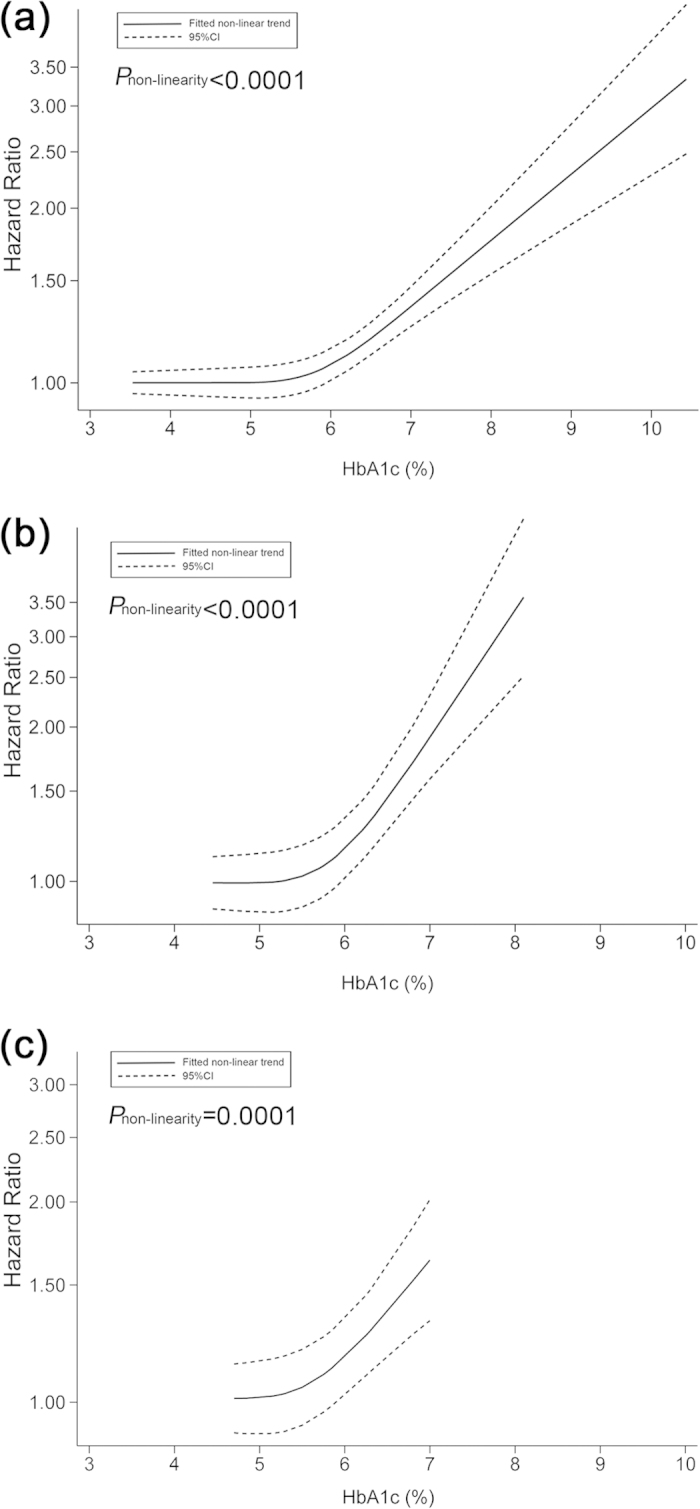
Non-linear dose–response analyses on the association of HbA_1c_ level with mortality from (**a**) all causes, (**b**) cardiovascular disease, and (**c**) cancer.

Using restricted cubic spline function, we found the evidence of non-linear associations between HbA_1c_ levels and risks of death from all causes (*P*_non-linearity_ < 0.0001) (Fig. 5a), CVD (*P*_non-linearity_ < 0.0001) (Fig. 5b) and cancer (*P*_non-linearity_ = 0.0001) (Fig. 5c). The curves were relatively flat when HbA_1c_ levels were less than approximately 5.7% (39 mmol/mol), and rose steeply thereafter. The curvilinear slope reached the maximal value at HbA_1c_ level of around 6.5% (48 mmol/mol). Similar increasing patterns were observed for CVD and cancer mortality.

### Subgroup and sensitivity analyses

The significant association between HbA_1c_ levels and all-cause mortality remained in all subgroups (see Supplementary Table S2). Pooled risk estimates from the random-effects model and fixed-effects model were virtually identical. Omitting a single study in turn did not significantly change the summary risk estimate of either all-cause or CVD mortality. Repeating meta-analyses according to various eligibility criteria did not change our pooled results, either (see Supplementary Table S3).

### Publication bias

There was no evidence of publication bias for any association as revealed by Begg’s test and Egger’s test (all *P* > 0.1).

## Discussion

The findings from the two-stage dose–response analyses suggested that in subjects without known diabetes, higher HbA_1c_ level is a predictor of increased all-cause and CVD mortality but not of cancer mortality. In subjects without diabetes, higher HbA_1c_ level is a predictor of increased CVD mortality only; in those with normal HbA_1c_ level, higher HbA_1c_ level is not a predictor of any studied mortality outcome.

We found evidence of a non-linear association between HbA_1c_ and mortality from all causes, CVD and cancer in this meta-analysis. The dose-response curves were relatively flat for HbA_1c_ less than around 5.7%, and rose steeply thereafter. This fact reveals a clear threshold effect for the association of HbA_1c_ levels with mortality. In addition, from the perspective of mortality benefit and health care burden, it suggests that the most appropriate HbA_1c_ level of initiating intervention is approximately 5.7%. Considering the essential requirement for HbA_1c_ to maintain normal metabolism of our body, it is reasonable that normal HbA_1c_ levels are not significantly associated with increased mortality, which was also clearly shown in our two-stage dose–response analyses on HbA_1c_ levels and mortality. However, mounting evidence shows that people are at high risk of some health conditions that could significantly increase mortality risk when HbA_1c_ level increases abnormally. For example, hyperglycemia measured by HbA_1c_ has been found to be associated with increased risks of stroke^39^, coronary heart disease^40^, colorectal cancer^41^, as well as hearing impairment^42^ in people without a history of diabetes.

In this study, our two-stage dose–response analyses did not find any significant associations with cancer mortality in the whole studied population as well as in subjects with HbA_1c_ < 6.5%. Considering limited studies and relatively wide CIs for risk estimates, the failure to detect significant associations was possibly caused by lack of power. In addition, if using a fixed-effects model to achieve data pooling, significant risk estimates could be obtained (for the whole studies population, HR = 1.03, 95% CI = 1.01–1.06, *P* < 0.01; for subjects with HbA_1c_ < 6.5%, HR = 1.03, 95% CI = 1.00–1.05, *P* = 0.03). With those considerations above, the results for cancer mortality in these two groups should be treated with caution, and more studies on HbA_1c_ and cancer mortality are needed.

Previous studies found that hyperglycemia measured by HbA_1c_ was significantly related to increased all-cause mortality in diabetic subjects^10^. Our study extended this association into subjects without known diabetes. The deleterious effect of hyperglycemia on mortality is biologically plausible. Ample evidence shows that hyperglycemia can result in oxidative stress (OS)^43^,^44^ through several potential mechanisms involving generation of reactive oxygen species, non-enzymatic glycation of proteins, and auto-oxidation of glucose^43^. OS then results in vascular endothelial dysfunction, which contributes to the presence of CVD^45^. OS can induce DNA damage^46^, protein carbonylation^47^, actions on signaling pathways^48^, possibly resulting in gene mutation or cell proliferation^48^. Therefore, OS is also a candidate contributor for cancer development^49^. Taken together, hyperglycemia-induced OS plays a major role in the underlying mechanisms for the association of hyperglycemia with increased mortality.

In the present meta-analysis, most of included studies ascertained non-diabetic status through self-reports. Consequently, our study population comprised a part of subjects with undiagnosed diabetes in the context of a large proportion of diabetic cases being not captured by self-reports^50^. Several epidemiological studies have demonstrated increased mortality in subjects with undiagnosed diabetes^51^,^52^. Considering the aforementioned facts, the increased all-cause and CVD mortality observed in the two-stage dose–response analyses might be resulted from inclusion of those with undiagnosed diabetes. Therefore, whether higher HbA_1c_ level is still predictive of increased mortality among subjects with HbA_1c_ < 6.5% is unclear. For addressing this critical concern, we repeated our meta-analyses by excluding subjects with undiagnosed diabetes. Corresponding results showed that the positive association with higher HbA_1c_ level remained for CVD mortality only, suggesting that higher HbA_1c_ level is still a predictor of increased CVD mortality but not of all-cause mortality in subjects with HbA_1c_ < 6.5%. The underlying reasons for the above phenomenon are unclear. A possible explanation is that the association between higher HbA_1c_ level and mortality from non-CVD is of extreme non-significance, making the contribution of CVD mortality to all-cause mortality insubstantial.

Several cross-sectional studies consistently indicate that HbA_1c_ levels increase with age in individuals without known diabetes^53^,^54^,^55^. Taking into account the above fact in combination with the dose–response patterns observed in our study, age is a possible effect modifier of the HbA_1c_-mortality association. However, the result of our subgroup analysis stratified by age did not provide evidence to support this hypothesis. Another candidate effect modifier is sex, considering that a sex difference in HbA_1c_ levels has been reported despite inconclusive results^53^,^56^. Of included studies, only 3^12^,^13^,^25^ investigated this potential effect modifier. Two of them^12^,^13^ consistently revealed no modification effect by sex for all-cause and CVD mortality, whereas the remaining one^25^ found that the significant positive association of HbA_1c_ levels with increased cancer mortality was only seen in women. Limited studies precluded our attempts to investigate this potential effect modifier through subgroup analysis^12^,^13^,^25^. Therefore, more studies are warranted to identify whether sex can modify the HbA_1c_-mortality association.

Our study has several limitations. First, all included studies measured HbA_1c_ level on a single occasion at baseline. Considering the within-subject variation of HbA_1c_ level and measurement error, our results might be subject to regression dilution bias^57^, indicating an underestimated effect size for the association of HbA_1c_ levels with mortality. Nonetheless, within-subject variation of HbA_1c_ in the healthy population has been found to be minimal^58^. Second, several included studies used death certificate to ascertain the causes of death^14^,^19^,^25^,^31^,^32^. Information from death certificate is inaccurate in some conditions^59^. Therefore, misclassification bias possibly affected our results on the associations between HbA_1c_ levels and risks of death from CVD and cancer. Third, although we extracted the most fully adjusted risk estimates, our results might be still biased by residual confounding. Nevertheless, the pooled results of maximally and minimally adjusted risk estimates are similar, indicating that the observed associations in our study cannot be explained predominantly by confounding. Fourth, we cannot calculate summary risk estimates for groups with undiagnosed diabetes as well as those with prediabetes because of diverse categorization across studies, although these data are important for medical practitioners. Fifth, our results may be influenced by language bias, because we restricted our search to studies published in English or Chinese. Nevertheless, it has been found that the effect of language restriction on the results of systematic reviews is non-significant^60^. Finally, although not indicated by both Begg’s test and Egger’s test, our results may be still driven by publication bias because above two tests have insufficient power when including limited studies. Moreover, exclusion of several studies where necessary data for dose–response analysis were not reported possibly results in publication bias.

In conclusion, higher HbA_1c_ level is associated with increased mortality from all causes, CVD, and cancer among subjects without known diabetes. However, this association is influenced by those with undiagnosed diabetes or prediabetes. Because of limited studies, the results in relation to cancer mortality should be treated with caution, and more studies are therefore warranted to investigate whether higher HbA_1c_ level is associated with increased cancer mortality.

## Additional Information

**How to cite this article**: Zhong, G.-C. *et al*. HbA_1c_ and Risks of All-Cause and Cause-Specific Death in Subjects without Known Diabetes: A Dose-Response Meta-Analysis of Prospective Cohort Studies. *Sci. Rep*. **6**, 24071; doi: 10.1038/srep24071 (2016).

## Supplementary Material

Supplementary Information

## References

[b1] Lenters-Westra E., Schindhelm R. K., Bilo H. J. & Slingerland R. J. Haemoglobin A1c: Historical overview and current concepts. Diabetes Res Clin Pract. 99, 75–84, doi: 10.1016/j.diabres.2012.10.007 (2013). 23176805

[b2] Paprott R. . Association Between Hemoglobin A1c and All-Cause Mortality: Results of the Mortality Follow-up of the German National Health Interview and Examination Survey 1998. Diabetes Care. 38, 249–256, doi: 10.2337/dc14-1787 (2015). 25414153

[b3] Committee I. E. International Expert Committee report on the role of the A1C assay in the diagnosis of diabetes. Diabetes Care. 32, 1327–1334, doi: 10.2337/dc09-9033 (2009). 19502545 PMC2699715

[b4] American Diabetes Association. Diagnosis and classification of diabetes mellitus. Diabetes Care. 37 Suppl 1, S81–90, doi: 10.2337/dc14-S081 (2014). 24357215

[b5] World Health Organization. Use of glycated haemoglobin (HbA_1c_) in the diagnosis of diabetes mellitus: abbreviated report of a WHO consultation. 2011. Geneva : World Health Organization (2013). 26158184

[b6] Pandey R. . Emerging trends in optical sensing of glycemic markers for diabetes monitoring. Trends in analytical chemistry: TRAC. 64, 100–108, doi: 10.1016/j.trac.2014.09.005 (2015). 25598563 PMC4295656

[b7] Wang B. & Anzai J.-i. Recent Progress in Electrochemical HbA_1c_ Sensors: A Review. Materials. 8, 1187–1203 (2015). 10.3390/ma8031187 PMC5455452 28787996

[b8] Chen C. . Recent advances in electrochemical glucose biosensors: a review. Rsc Advances. 3, 4473–4491 (2013).

[b9] Spegazzini N. . Spectroscopic approach for dynamic bioanalyte tracking with minimal concentration information. Scientific Reports. 4, 7013, doi: 10.1038/srep07013 (2014). 25388455 PMC4894421

[b10] Zhang Y., Hu G., Yuan Z. & Chen L. Glycosylated hemoglobin in relationship to cardiovascular outcomes and death in patients with type 2 diabetes: a systematic review and meta-analysis. PLoS One. 7, e42551, doi: 10.1371/journal.pone.0042551 (2012). 22912709 PMC3415427

[b11] Arnold L. W. & Wang Z. The HbA_1c_ and all-cause mortality relationship in patients with type 2 diabetes is J-shaped: a meta-analysis of observational studies. The review of diabetic studies: RDS. 11, 138–152, doi: 10.1900/rds.2014.11.138 (2014). 25396402 PMC4310064

[b12] Bancks M. P. . Glycated hemoglobin and all-cause and cause-specific mortality in Singaporean Chinese without diagnosed diabetes: The Singapore Chinese Health Study. Diabetes Care. 37, 3180–3187 (2014). 25216509 10.2337/dc14-0390 PMC4237977

[b13] Sakurai M. . HbA_1c_ and the risks for all-cause and cardiovascular mortality in the general Japanese population: NIPPON DATA90. Diabetes Care. 36, 3759–3765, doi: 10.2337/dc12-2412 (2013). 23877989 PMC3816883

[b14] Chonchol M. . Glycosylated hemoglobin and the risk of death and cardiovascular mortality in the elderly. Nutrition, metabolism, and cardiovascular diseases: NMCD. 20, 15–21, doi: 10.1016/j.numecd.2009.02.007 (2010). PMC2888268 19364638

[b15] Saydah S., Tao M., Imperatore G. & Gregg E. GHb level and subsequent mortality among adults in the U.S. Diabetes Care. 32, 1440–1446, doi: 10.2337/dc09-0117 (2009). 19401445 PMC2713636

[b16] Santos-Oliveira R. . Haemoglobin A1c levels and subsequent cardiovascular disease in persons without diabetes: a meta-analysis of prospective cohorts. Diabetologia. 54, 1327–1334, doi: 10.1007/s00125-011-2078-8 (2011). 21340623

[b17] Singer D. E., Nathan D. M., Anderson K. M., Wilson P. W. & Evans J. C. Association of HbA_1c_ with prevalent cardiovascular disease in the original cohort of the Framingham Heart Study. Diabetes. 41, 202–208 (1992). 1733810 10.2337/diab.41.2.202

[b18] Khaw K. T. . Association of hemoglobin A1c with cardiovascular disease and mortality in adults: the European prospective investigation into cancer in Norfolk. Ann Intern Med. 141, 413–420 (2004). 15381514 10.7326/0003-4819-141-6-200409210-00006

[b19] Gao L., Matthews F. E., Sargeant L. A. & Brayne C. An investigation of the population impact of variation in HbA 1clevels in older people in England and Wales: From a population based multi-centre longitudinal study. BMC Public Health. 8 (2008). 10.1186/1471-2458-8-54 PMC2275259 18267013

[b20] de Vegt F. . Hyperglycaemia is associated with all-cause and cardiovascular mortality in the Hoorn population: the Hoorn Study. Diabetologia. 42, 926–931, doi: 10.1007/s001250051249 (1999). 10491751

[b21] Corpus R. A., O’Neill W. W., Dixon S. R., Timmis G. C. & Devlin W. H. Relation of hemoglobin A1c to rate of major adverse cardiac events in nondiabetic patients undergoing percutaneous coronary revascularization. The American journal of cardiology. 92, 1282–1286 (2003). 14636904 10.1016/j.amjcard.2003.08.008

[b22] Moher D., Liberati A., Tetzlaff J. & Altman D. G. Preferred reporting items for systematic reviews and meta-analyses: the PRISMA statement. BMJ (Clinical research ed.). 339, b2535, doi: 10.1136/bmj.b2535 (2009). PMC2714657 19622551

[b23] Hanas R. & John G. 2010 consensus statement on the worldwide standardization of the hemoglobin A1C measurement. Diabetes Care. 33, 1903–1904, doi: 10.2337/dc10-0953 (2010). 20519665 PMC2909083

[b24] Levitan E. B. . HbA_1c_ measured in stored erythrocytes and mortality rate among middle-aged and older women. Diabetologia. 51, 267–275, doi: 10.1007/s00125-007-0882-y (2008). 18043905 PMC2757266

[b25] Joshu C. E. . Glycated hemoglobin and cancer incidence and mortality in the Atherosclerosis in Communities (ARIC) Study, 1990–2006. International Journal of Cancer. 131, 1667–1677 (2012). 22161730 10.1002/ijc.27394 PMC3906204

[b26] Higgins J. P., Thompson S. G., Deeks J. J. & Altman D. G. Measuring inconsistency in meta-analyses. BMJ (Clinical research ed.). 327, 557–560, doi: 10.1136/bmj.327.7414.557 (2003). PMC192859 12958120

[b27] Higgins J. P. & Thompson S. G. Quantifying heterogeneity in a meta-analysis. Statistics in medicine. 21, 1539–1558, doi: 10.1002/sim.1186 (2002). 12111919

[b28] Orsini N., Bellocco R. & Greenland S. Generalized least squares for trend estimation of summarized dose–response data. The Stata Journal. 6(1), 40–57 (2006).

[b29] Hartemink N., Boshuizen H. C., Nagelkerke N. J., Jacobs M. A. & van Houwelingen H. C. Combining risk estimates from observational studies with different exposure cutpoints: a meta-analysis on body mass index and diabetes type 2. Am J Epidemiol. 163, 1042–1052, doi: 10.1093/aje/kwj141 (2006). 16611666

[b30] Hamling J., Lee P., Weitkunat R. & Ambuhl M. Facilitating meta-analyses by deriving relative effect and precision estimates for alternative comparisons from a set of estimates presented by exposure level or disease category. Statistics in medicine. 27, 954–970, doi: 10.1002/sim.3013 (2008). 17676579

[b31] Pfister R., Sharp S. J., Luben R., Khaw K. T. & Wareham N. J. No evidence of an increased mortality risk associated with low levels of glycated haemoglobin in a non-diabetic UK population. Diabetologia. 54, 2025–2032 (2011). 21584793 10.1007/s00125-011-2162-0

[b32] Nakanishi S., Yamada M., Hattori N. & Suzuki G. Relationship between HbA(1)c and mortality in a Japanese population. Diabetologia. 48, 230–234, doi: 10.1007/s00125-004-1643-9 (2005). 15650819

[b33] Orsini N., Li R., Wolk A., Khudyakov P. & Spiegelman D. Meta-analysis for linear and nonlinear dose-response relations: examples, an evaluation of approximations, and software. Am J Epidemiol. 175, 66–73, doi: 10.1093/aje/kwr265 (2012). 22135359 PMC3244608

[b34] Desquilbet L. & Mariotti F. Dose-response analyses using restricted cubic spline functions in public health research. Statistics in medicine. 29, 1037–1057, doi: 10.1002/sim.3841 (2010). 20087875

[b35] Begg C. B. & Mazumdar M. Operating characteristics of a rank correlation test for publication bias. Biometrics. 50, 1088–1101 (1994). 7786990

[b36] Egger M., Davey Smith G., Schneider M. & Minder C. Bias in meta-analysis detected by a simple, graphical test. BMJ (Clinical research ed.). 315, 629–634 (1997). 10.1136/bmj.315.7109.629 PMC2127453 9310563

[b37] Selvin E. . Glycated hemoglobin, diabetes, and cardiovascular risk in nondiabetic adults. New England Journal of Medicine. 362, 800–811 (2010). 20200384 10.1056/NEJMoa0908359 PMC2872990

[b38] Skriver M. V., Borch-Johnsen K., Lauritzen T. & Sandbaek A. HbA_1c_ as predictor of all-cause mortality in individuals at high risk of diabetes with normal glucose tolerance, identified by screening: a follow-up study of the Anglo-Danish-Dutch Study of Intensive Treatment in People with Screen-Detected Diabetes in Primary Care (ADDITION), Denmark. Diabetologia. 53, 2328–2333, doi: 10.1007/s00125-010-1867-9 (2010). 20697688

[b39] Selvin E., Rawlings A. M., Bergenstal R. M., Coresh J. & Brancati F. L. No racial differences in the association of glycated hemoglobin with kidney disease and cardiovascular outcomes. Diabetes Care. 36, 2995–3001, doi: 10.2337/dc12-2715 (2013). 23723353 PMC3781554

[b40] Sarwar N. . Markers of dysglycaemia and risk of coronary heart disease in people without diabetes: Reykjavik prospective study and systematic review. PLoS Med. 7, e1000278, doi: 10.1371/journal.pmed.1000278 (2010). 20520805 PMC2876150

[b41] Rinaldi S. . Glycosylated hemoglobin and risk of colorectal cancer in men and women, the European prospective investigation into cancer and nutrition. Cancer epidemiology, biomarkers & prevention: a publication of the American Association for Cancer Research, cosponsored by the American Society of Preventive Oncology. 17, 3108–3115, doi: 10.1158/1055-9965.epi-08-0495 (2008). 18990751

[b42] Michikawa T., Mizutari K., Saito H., Takebayashi T. & Nishiwaki Y. Glycosylated hemoglobin level is associated with hearing impairment in older Japanese: the Kurabuchi Study. J Am Geriatr Soc. 62, 1231–1237, doi: 10.1111/jgs.12906 (2014). 25039656

[b43] Blaak E. E. . Impact of postprandial glycaemia on health and prevention of disease. Obesity reviews: an official journal of the International Association for the Study of Obesity. 13, 923–984, doi: 10.1111/j.1467-789X.2012.01011.x (2012). 22780564 PMC3494382

[b44] Rains J. L. & Jain S. K. Oxidative stress, insulin signaling, and diabetes. Free radical biology & medicine. 50, 567–575, doi: 10.1016/j.freeradbiomed.2010.12.006 (2011). 21163346 PMC3557825

[b45] Mah E. & Bruno R. S. Postprandial hyperglycemia on vascular endothelial function: mechanisms and consequences. Nutrition research (New York, N.Y.). 32, 727–740, doi: 10.1016/j.nutres.2012.08.002 (2012). 23146769

[b46] Loft S. . Biomarkers of oxidative damage to DNA and repair. Biochem Soc Trans. 36, 1071–1076, doi: 10.1042/bst0361071 (2008). 18793191

[b47] Rao R. S. & Moller I. M. Pattern of occurrence and occupancy of carbonylation sites in proteins. Proteomics. 11, 4166–4173, doi: 10.1002/pmic.201100223 (2011). 21919202

[b48] Mates J. M., Segura J. A., Alonso F. J. & Marquez J. Intracellular redox status and oxidative stress: implications for cell proliferation, apoptosis, and carcinogenesis. Archives of toxicology. 82, 273–299, doi: 10.1007/s00204-008-0304-z (2008). 18443763

[b49] Thanan R. . Oxidative stress and its significant roles in neurodegenerative diseases and cancer. Int J Mol Sci. 16, 193–217, doi: 10.3390/ijms16010193 (2015). 25547488 PMC4307243

[b50] Leong A., Dasgupta K., Chiasson J. L. & Rahme E. Estimating the population prevalence of diagnosed and undiagnosed diabetes. Diabetes Care. 36, 3002–3008, doi: 10.2337/dc12-2543 (2013). 23656982 PMC3781536

[b51] Valdes S., Botas P., Delgado E. & Diaz Cadorniga F. Mortality risk in spanish adults with diagnosed diabetes, undiagnosed diabetes or pre-diabetes. The Asturias study 1998-2004. Revista espanola de cardiologia. 62, 528–534 (2009). 19406067 10.1016/s1885-5857(09)71835-3

[b52] Wild S. H., Smith F. B., Lee A. J. & Fowkes F. G. Criteria for previously undiagnosed diabetes and risk of mortality: 15-year follow-up of the Edinburgh Artery Study cohort. Diabetic medicine: a journal of the British Diabetic Association. 22, 490–496, doi: 10.1111/j.1464-5491.2004.01433.x (2005). 15787678

[b53] Yang Y. C., Lu F. H., Wu J. S. & Chang C. J. Age and sex effects on HbA_1c_. A study in a healthy Chinese population. Diabetes Care. 20, 988–991 (1997). 9167111 10.2337/diacare.20.6.988

[b54] Yates A. P. & Laing I. Age-related increase in haemoglobin A1c and fasting plasma glucose is accompanied by a decrease in beta cell function without change in insulin sensitivity: evidence from a cross-sectional study of hospital personnel. Diabetic medicine: a journal of the British Diabetic Association. 19, 254–258 (2002). 11918628 10.1046/j.1464-5491.2002.00644.x

[b55] Davidson M. B. & Schriger D. L. Effect of age and race/ethnicity on HbA_1c_ levels in people without known diabetes mellitus: implications for the diagnosis of diabetes. Diabetes Res Clin Pract. 87, 415–421, doi: 10.1016/j.diabres.2009.12.013 (2010). 20061043

[b56] Bae J. C. . Hemoglobin A1c values are affected by hemoglobin level and gender in non-anemic Koreans. J Diabetes Investig. 5, 60–65, doi: 10.1111/jdi.12123 (2014). PMC4025240 24843738

[b57] MacMahon S. . Blood pressure, stroke, and coronary heart disease. Part 1, Prolonged differences in blood pressure: prospective observational studies corrected for the regression dilution bias. Lancet. 335, 765–774 (1990). 1969518 10.1016/0140-6736(90)90878-9

[b58] Lenters-Westra E., Roraas T., Schindhelm R. K., Slingerland R. J. & Sandberg S. Biological variation of hemoglobin A1c: consequences for diagnosing diabetes mellitus. Clin Chem. 60, 1570–1572, doi: 10.1373/clinchem.2014.227983 (2014). 25248570

[b59] Kircher T., Nelson J. & Burdo H. The autopsy as a measure of accuracy of the death certificate. The New England journal of medicine. 313, 1263–1269, doi: 10.1056/nejm198511143132005 (1985). 4058507

[b60] Moher D., Pham B., Lawson M. L. & Klassen T. P. The inclusion of reports of randomised trials published in languages other than English in systematic reviews. Health technology assessment (Winchester, England). 7, 1–90 (2003). 10.3310/hta7410 14670218

