# Supplementary material for: HbA1c and Risks of All-Cause and Cause-Specific Death in Subjects without Known Diabetes: A Dose-Response Meta-Analysis of Prospective Cohort Studies
Source: Sci Rep. 2016 Apr 5;6:24071. doi: 10.1038/srep24071 (PMC4820688; doi:10.1038/srep24071)
Supplement: Supplementary Information [file srep24071-s1.pdf]

HbA_{1c} and Risks of All-Cause and Cause-Specific Death in Subjects without Known Diabetes: A Dose-Response Meta-Analysis of Prospective Cohort Studies

Guo-Chao Zhong, Ming-Xin Ye, Jia-Hao Cheng, Yong Zhao, Jian-Ping Gong

Supplementary List S1 Search Strategy

PubMed

#1 (death[Title/Abstract]) OR mortality[Title/Abstract]

#2 ((((((((((((((glycated hemoglobin[Title/Abstract]) OR glycosylated hemoglobin[Title/Abstract]) OR glycosylated haemoglobin[Title/Abstract]) OR glycated haemoglobin[Title/Abstract]) OR hemoglobin A1[Title/Abstract]) OR hemoglobin A1c[Title/Abstract]) OR HbA1[Title/Abstract]) OR HbA1c[Title/Abstract]) OR Haemoglobin A1c[Title/Abstract]) OR Haemoglobin A1[Title/Abstract]) OR hemoglobin a, glycosylated[MeSH Terms]) OR A1c[Title/Abstract]) OR glycaemic control[Title/Abstract]) OR glycemic control[Title/Abstract]) OR A1[Title/Abstract]

#3 #1 AND #2

#4 review[Publication Type]

#5 letter[Publication Type]

#6 #4 OR #5

#7 #3 NOT #6

#8 (English[lang] OR Chinese[lang])

#9 #7 AND #8

EMBASE

#1 death:ab,ti AND ([article]/lim OR [article in press]/lim OR [conference abstract]/lim OR [conference paper]/lim) AND ([chinese]/lim OR [english]/lim) AND [embase]/lim

#2 mortality:ab,ti AND ([article]/lim OR [article in press]/lim OR [conference abstract]/lim OR [conference paper]/lim) AND ([chinese]/lim OR [english]/lim) AND [embase]/lim

#3 #1 OR #2

#4 glycated AND hemoglobin:ab,ti AND ([article]/lim OR [article in press]/lim OR [conference abstract]/lim OR [conference paper]/lim) AND ([chinese]/lim OR [english]/lim) AND [embase]/lim

#5 glycosylated AND hemoglobin:ab,ti AND ([article]/lim OR [article in press]/lim OR [conference abstract]/lim OR [conference paper]/lim)

AND ([chinese]/lim OR [english]/lim) AND [embase]/lim

#6 glycosylated AND haemoglobin:ab,ti AND ([article]/lim OR [article in press]/lim OR [conference abstract]/lim OR [conference paper]/lim) AND ([chinese]/lim OR [english]/lim) AND [embase]/lim

#7 glycated AND haemoglobin:ab,ti AND ([article]/lim OR [article in press]/lim OR [conference abstract]/lim OR [conference paper]/lim) AND ([chinese]/lim OR [english]/lim) AND [embase]/lim

#8 'hemoglobin'/exp OR hemoglobin AND a1:ab,ti AND ([article]/lim OR [article in press]/lim OR [conference abstract]/lim OR [conference paper]/lim) AND ([chinese]/lim OR [english]/lim) AND [embase]/lim

#9 'hemoglobin'/exp OR hemoglobin AND a1c:ab,ti AND ([article]/lim OR [article in press]/lim OR [conference abstract]/lim OR [conference paper]/lim) AND ([chinese]/lim OR [english]/lim) AND [embase]/lim

#10 hba1:ab,ti AND ([article]/lim OR [article in press]/lim OR [conference abstract]/lim OR [conference paper]/lim) AND ([chinese]/lim OR [english]/lim) AND [embase]/lim

#11 hba1c:ab,ti AND ([article]/lim OR [article in press]/lim OR [conference abstract]/lim OR [conference paper]/lim) AND ([chinese]/lim OR [english]/lim) AND [embase]/lim

#12 'haemoglobin'/exp OR haemoglobin AND a1c:ab,ti AND ([article]/lim OR [article in press]/lim OR [conference abstract]/lim OR [conference paper]/lim) AND ([chinese]/lim OR [english]/lim) AND [embase]/lim

#13 'haemoglobin'/exp OR haemoglobin AND a1:ab,ti AND ([article]/lim OR [article in press]/lim OR [conference abstract]/lim OR [conference paper]/lim) AND ([chinese]/lim OR [english]/lim) AND [embase]/lim

#14 a1c:ab,ti AND ([article]/lim OR [article in press]/lim OR [conference abstract]/lim OR [conference paper]/lim) AND ([chinese]/lim OR [english]/lim) AND [embase]/lim

#15 glycaemic AND control:ab,ti AND ([article]/lim OR [article in press]/lim OR [conference abstract]/lim OR [conference paper]/lim) AND ([chinese]/lim OR [english]/lim) AND [embase]/lim

#16 glycemic AND control:ab,ti AND ([article]/lim OR [article in press]/lim OR [conference abstract]/lim OR [conference paper]/lim) AND ([chinese]/lim OR [english]/lim) AND [embase]/lim

#17 a1:ab,ti AND ([article]/lim OR [article in press]/lim OR [conference abstract]/lim OR [conference paper]/lim) AND ([chinese]/lim OR [english]/lim) AND [embase]/lim 25,400

#18 #4 OR #5 OR #6 OR #7 OR #8 OR #9 OR #10 OR #11 OR #12 OR #13 OR #14 OR #15 OR #16 OR #17

#19 #3 AND # 18

Supplementary Table S1. Characteristics of included studies regarding HbA_{1c} levels and mortality

Source/ Publication year	Study location	Mean age , years	Sample size	Mean follow-up, years	Assessment of non-diabetic status	Assessment of outcome	Method of measuring HbA_{1c}	Adjustment factors
Paprott et al [2] 2015	Germany	44.7	T: 5986<br>M: 2932<br>W: 3054	11.6	Self-report	Death registry	HPLC	Age, sex, alcohol, BMI, education, history of hypertension or hyperlipidemia, history of myocardial infarction, stroke or cancer, smoking, sport activity, waist circumference
Bancks et al [12] 2014	Singapore	62.3	T: 7388<br>M: 3562<br>W: 3826	10.1	Self-report	Linkage to the nationwide registry of birth and death	HPLC	Age, sex, alcohol, BMI, dialect, education, hypertension, interview year, smoking
Sakurai et al [13] 2013	Japan	52.0	T: 6929<br>M: 2868<br>W: 4061	14.0	Self-report	Data from the National Vital Statistics database	HPLC	Age, sex, alcohol, BMI, habitual exercise, HDL cholesterol, medical treatment for hypertension and dyslipidemia, smoking, systolic blood pressure,

								total cholesterol
Joshu et al [25] 2012	USA	56.7	T: 11668<br>M: 5291<br>W: 6377	13.2	Self-report	Death certificate	HPLC	Age, BMI, cigarette-years smoked, education, postmenopausal hormone use (women only), race, smoking, study site, waist circumference
Pfister et al [31] 2011	UK	59.2	T: 17196<br>M: 7482<br>W: 9714	11.2	Self-report	Death certificate	HPLC	Age, sex, alcohol, physical activity, smoking, systolic blood pressure, total cholesterol, waist-to-hip ratio
Skriver et al [38] 2010	Denmark	58.1	T: 15634<br>M: 8010<br>W: 7624	6.6	Diagnostic test	Linkage to the nationwide Danish Civil Registration System	HPLC	Age, sex, BMI, cancer, cerebrovascular disease, ischemic heart disease, smoking, systolic blood pressure
Selvin et al [37] 2010	USA	56.7	T: 11092<br>M: 4692<br>W: 6400	13.2	Self-report	Death certificate	HPLC	Age, sex, alcohol, baseline fasting glucose level, BMI, education, family history of diabetes, hypertension, HDL, LDL, log-transformed triglyceride level, physical-activity index score, race, smoking,

								waist-to-hip ratio
Chonchol et al[14] 2010	USA	72.0	T: 810<br>M: 330<br>W: 480	14.2	Anti-diabetic medication use or diagnostic test	Obituary, medical record, death certificate, health care utilization database	Affinity column method	Age, sex, BMI, chronic kidney disease, hypertension, LDL cholesterol, race, smoking
Saydah et al [15] 2009	USA	47.6	T: 17570<br>M: 9709<br>W: 7861	8.4	Self-report	Linkage to the National Death Index	HPLC	Sex, BMI, education, HDL cholesterol, race, smoking, systolic blood pressure
Levitan et al [24] 2008	USA	54.6	T: 26549<br>M: 0<br>W:26549	10.2 [a]	Self-report	Mailed questionnaire, national death index, medical record	TIA	Age, BMI, HDL cholesterol, history of hypertension, hsCRP, LDL cholesterol, multivitamin use, postmenopausal hormone use (women only), quintile of triacylglycerol, smoking, strenuous exercise
Gao et al [19] 2008	UK	78.7	T: 1040<br>M: 438<br>W: 602	5.0	Self-report	Death certificate	HPLC	Age, sex
Nakanish et al [32] 2005	Japan	67.6	T: 3332<br>M: 994<br>W: 2338	8.8	Self-report	Death certificate	HPLC	Age, sex, A-bomb kerma dose, BMI, cholesterol, drinking, smoking, systolic blood pressure

Abbreviations: BMI, body mass index; CVD, cardiovascular disease; HDL, high-density lipoprotein; HPLC, high performance liquid

chromatography; hsCRP , high-sensitivity C-reactive protein; LDL, low-density lipoprotein; M, men; T, total; TIA, turbidimetric immunoinhibition assay; W, women.

[a] Median value.

Supplementary Table S2. Subgroup analyses regarding HbA_{1c} levels and all-cause mortality in the whole studied population

Subgroup	Number of studies	HR (95% CI)	I^2 (%)	$P_{heterogeneity}$	$P_{interaction}$
All studies	11	1.03 (1.02, 1.04)	0.0	0.46	-
Age					
≥65y	3	1.03 (1.01, 1.05)	9.1	0.33	0.69
<65y	8	1.03 (1.02, 1.04)	4.9	0.39	
Follow-up duration					
≥10y	7	1.03 (1.02, 1.04)	4.4	0.39	0.70
<10y	4	1.03 (1.01, 1.05)	7.5	0.36	
Sample size					
≥10,000	5	1.03 (1.02, 1.05)	37.3	0.17	0.64
<10,000	6	1.03 (1.02, 1.04)	0.0	0.69	
Study location					
Europe	4	1.03 (1.02, 1.04)	0.0	0.83	0.86
North America	4	1.03 (1.01, 1.05)	63.4	0.04	
Asia	3	1.04 (1.02, 1.05)	0.0	0.84	

Abbreviations: CI, confidence interval; HR, hazard ratio.

Supplementary Table S3. Sensitivity analyses regarding HbA1c levels and all-cause and cardiovascular disease mortality

Categories	All-cause mortality			Cardiovascular disease mortality		
	No. of studies	HR (95% CI)	I^2 (%)	No. of studies	HR（95% CI)	I^2 (%)

Random-effects model	11	1.03 (1.02, 1.04)	0.0	6	1.05 (1.03, 1.07)	10.7
Fixed-effects model	11	1.03 (1.02, 1.04)	0.0	6	1.05 (1.03, 1.06)	10.7
Participants including men and women	10	1.03 (1.02, 1.04)	6.3	6	1.05 (1.03, 1.07)	10.7
HPLC as method of measuring HbA_{1c}	9	1.03 (1.02, 1.04)	0.0	5	1.05 (1.03, 1.07)	0.0
Assessment of non-diabetic status through self-report	9	1.03 (1.02, 1.04)	0.0	5	1.05 (1.03, 1.07)	0.0
Measuring HbA_{1c} rather than glycated hemoglobin	7	1.03 (1.02, 1.05)	0.0	4	1.07 (1.04, 1.10)	0.0
Person-years directly reported by authors	7	1.03 (1.02, 1.05)	2.3	3	1.06 (1.01, 1.10)	54.1

Abbreviations: CI, confidence interval; HPLC, high performance liquid chromatography; HR, hazard ratio.
